# 2-(2-Chloro­phen­yl)-3-(3,4-dimeth­oxy­phen­yl)quinoxaline

**DOI:** 10.1107/S1600536810024864

**Published:** 2010-07-31

**Authors:** Stefanie A. Cantalupo, Guy Crundwell, Neil Glagovich

**Affiliations:** a Department of Chemistry, Central Connecticut State University, New Britain, CT 06053, USA

## Abstract

The title compound, C_22_H_17_ClN_2_O_2_, was synthesized by the condensation reaction between 1,2-phenyl­enediamine and 2-chloro-3′,4′-dimeth­oxy­benzil in boiling acetic acid. The chloro­phenyl and dimeth­oxy­phenyl rings make dihedral angles of 78.45 (5) and 35.60 (4)°, respectively, with the quinoxaline unit.

## Related literature

*N*-heterocyclic aromatic compounds are of current inter­est as ligands in one- and two-dimensional coordination polymers with silver, see: Fitchett & Steel (2006 ▶). The quinoxaline moiety yields a wide variety of potential bidentate bridges in polymeric networks with silver, see: Patra *et al.* (2007 ▶). For the synthesis and characterization of quinoxalines, see: Crundwell & Stacy (2005 ▶), of benzo[*g*]quinoxalines, see: Cantalupo *et al.* (2006 ▶) and of pyrazino­[2,3-*g*]quinoxalines, see: Bellizzi *et al.* (2006 ▶).
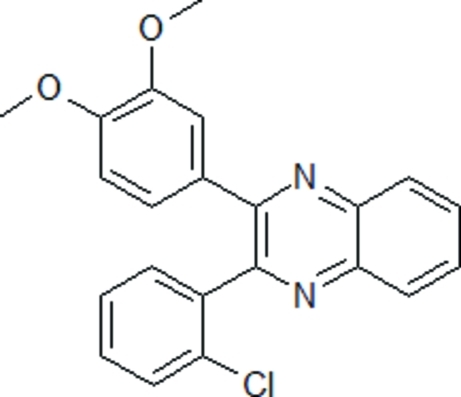


## Experimental

### 

#### Crystal data

C_22_H_17_ClN_2_O_2_
*M* *_r_* = 376.83
Monoclinic, 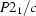

*a* = 14.6741 (13) Å
*b* = 7.9731 (7) Å
*c* = 21.6996 (17) Å
β = 132.560 (6)°
*V* = 1870.0 (3) Å^3^
*Z* = 4
Mo *K*α radiation
μ = 0.22 mm^−1^
*T* = 293 K
0.42 × 0.24 × 0.19 mm

#### Data collection

Oxford Diffraction Xcalibur Sapphire3 diffractometer
Absorption correction: multi-scan (*CrysAlis PRO*; Oxford Diffraction, 2009 ▶) *T*_min_ = 0.699, *T*_max_ = 1.000
46880 measured reflections
7159 independent reflections
4223 reflections with *I* > 2σ(*I*)
*R*_int_ = 0.051

#### Refinement

*R*[*F* ^2^ > 2σ(*F* ^2^)] = 0.069
*wR*(*F* ^2^) = 0.202
*S* = 1.03
7159 reflections
246 parameters
H-atom parameters constrained
Δρ_max_ = 0.41 e Å^−3^
Δρ_min_ = −0.37 e Å^−3^

### 

Data collection: *CrysAlis CCD* (Oxford Diffraction, 2009 ▶); cell refinement: *CrysAlis RED* (Oxford Diffraction, 2009 ▶); data reduction: *CrysAlis RED*; program(s) used to solve structure: *SHELXS97* (Sheldrick, 2008 ▶); program(s) used to refine structure: *SHELXL97* (Sheldrick, 2008 ▶); molecular graphics: *ORTEP-3* (Farrugia, 1997 ▶); software used to prepare material for publication: *SHELXTL* (Sheldrick, 2008 ▶).

## Supplementary Material

Crystal structure: contains datablocks global, I. DOI: 10.1107/S1600536810024864/ds2038sup1.cif

Structure factors: contains datablocks I. DOI: 10.1107/S1600536810024864/ds2038Isup2.hkl

Additional supplementary materials:  crystallographic information; 3D view; checkCIF report

## Figures and Tables

**Fig. 1.**
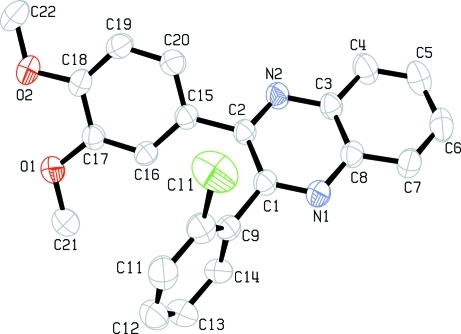
A view of the title compound (Farrugia, 1997). Displacement ellipsoids are drawn at the 50% probability level.

## supplementary crystallographic information

### Comment

N-heterocyclic aromatic compounds are of current interest as ligands in one- and two-dimensional coordination polymers with silver (Fitchett *et al.*, 2006). The quinoxaline moiety specifically is an enticing aromatic heterocycle since it is readily formed *via* condensation reactions between diketones and di- or tetra-amines and it yields a wide variety of potential bidentate bridges in polymeric networks with silver (Patra *et al.*, 2007).

The Crundwell lab has synthesized and characterized many quinoxalines (Crundwell *et al.*, 2005), benzo[*g*]quinoxalines (Cantalupo *et al.*, 2006), and pyrazino[2,3-*g*]quinoxalines (Bellizzi *et al.*, 2006) as potential metal ligands. The title compound was formed by the condensation of two commercial products: 1,2-phenylenediamine and 2-chloro-3',4'-dimethoxybenzil. The resulting quinoxaline had bond lengths that fell within expectated values and had ring torsion angles of 78.45 (5)° and 35.60 (4)° with respect to the planar quinoxaline moiety.

### Experimental

To a 100 mL round bottom flask equipped with a Hickman still and a reflux condenser was combined 0.1556 g (1.46 mmol) 1,2-phenylenediamine and 0.4465 g (1.46 mmol) of 2-chloro-3',4'-dimethoxybenzil in 50 mL of concentrated acetic acid.

The mixture was refluxed for 16 h and the resulting solution was chilled then filtered to produce a pale yellow solid. The solid was recrystallized from a 50/50 mixture of toluene and ethanol to yield 0.312 g of 2-(2-chlorophenyl)-3-(3,4-dimethoxyphenyl)-quinoxaline (56.5%).

mp 407.8; ^1^H NMR (300 MHz, CDCl_3_): δ 8.193 (ddd, 2H, J = 7.2 Hz, J = 2.4 Hz, J = 0.6 Hz), 7.797 (ddt, 2H, J = 7.2 Hz, J = 6.9 Hz, J = 2.4 Hz), 7.528 (ddd, 1H, J = 5.7 Hz, J = 2.4 Hz, J = 1.8 Hz), 7.372 (m, 3H), 7.210 (dd, 1H, J = 8.4 Hz, J = 2.1 Hz), 7.014 (d, 1H, J = 2.1 Hz), 6.818 (d, 1H, J = 8.4 Hz), 3.878 (s, 3H), 3.657 (s, 3H); ^13^C NMR (300 MHz, CDCl_3_): δ 153.15, 151.93, 149.77, 148.31, 141.76, 140.54, 139.02, 133.11, 131.27, 130.83, 130.39, 130.04, 129.88, 129.74, 129.22, 129.19, 127.13, 122.69, 112.43, 110.76, 55.84, 55.62.

### Refinement

Hydrogen atoms were included in calculated positions with a C—H distance of 0.95 Å and were included in the refinement in riding motion approximation with *U*_iso_ = 1.2*U*_eq_ of the carrier atom.

### Crystal data

**Table:**

C_22_H_17_ClN_2_O_2_	*F*(000) = 784
*M**_r_* = 376.83	*D*_x_ = 1.338 Mg m^−^^3^
Monoclinic, *P*2_1_/*c*	Melting point: 407.8 K
Hall symbol: -P 2ybc	Mo *K*α radiation, λ = 0.71073 Å
*a* = 14.6741 (13) Å	Cell parameters from 11257 reflections
*b* = 7.9731 (7) Å	θ = 4.3–34.1°
*c* = 21.6996 (17) Å	µ = 0.22 mm^−^^1^
β = 132.560 (6)°	*T* = 293 K
*V* = 1870.0 (3) Å^3^	Block, yellow
*Z* = 4	0.42 × 0.24 × 0.19 mm

### Data collection

**Table:**

Oxford Diffraction Xcalibur Sapphire3 diffractometer	7159 independent reflections
Radiation source: Enhance (Mo) X-ray Source	4223 reflections with *I* > 2σ(*I*)
graphite	*R*_int_ = 0.051
Detector resolution: 16.1790 pixels mm^-1^	θ_max_ = 33.9°, θ_min_ = 4.4°
ω scans	*h* = −22→22
Absorption correction: multi-scan (*CrysAlis PRO*; Oxford Diffraction, 2009)	*k* = −12→12
*T*_min_ = 0.699, *T*_max_ = 1.000	*l* = −33→33
46880 measured reflections	

### Refinement

**Table:**

Refinement on *F*^2^	Primary atom site location: structure-invariant direct methods
Least-squares matrix: full	Secondary atom site location: difference Fourier map
*R*[*F*^2^ > 2σ(*F*^2^)] = 0.069	Hydrogen site location: inferred from neighbouring sites
*wR*(*F*^2^) = 0.202	H-atom parameters constrained
*S* = 1.02	*w* = 1/[σ^2^(*F*_o_^2^) + (0.0955*P*)^2^ + 0.4554*P*] where *P* = (*F*_o_^2^ + 2*F*_c_^2^)/3
7159 reflections	(Δ/σ)_max_ < 0.001
246 parameters	Δρ_max_ = 0.41 e Å^−^^3^
0 restraints	Δρ_min_ = −0.37 e Å^−^^3^

### Special details

**Table:**

Experimental. Hydrogen atoms were included in calculated positions with a C—H distance of 0.95 Å and were included in the refinement in riding motion approximation with *U*_iso_ = 1.2*U*_eq_ of the carrier atom.CrysAlisPro (Oxford Diffraction Ltd., 2009) Empirical absorption correction using spherical harmonics, implemented in SCALE3 ABSPACK scaling algorithm.
Geometry. All e.s.d.'s (except the e.s.d. in the dihedral angle between two l.s. planes) are estimated using the full covariance matrix. The cell e.s.d.'s are taken into account individually in the estimation of e.s.d.'s in distances, angles and torsion angles; correlations between e.s.d.'s in cell parameters are only used when they are defined by crystal symmetry. An approximate (isotropic) treatment of cell e.s.d.'s is used for estimating e.s.d.'s involving l.s. planes.
Refinement. Refinement of *F*^2^ against ALL reflections. The weighted *R*-factor *wR* and goodness of fit *S* are based on *F*^2^, conventional *R*-factors *R* are based on *F*, with *F* set to zero for negative *F*^2^. The threshold expression of *F*^2^ > σ(*F*^2^) is used only for calculating *R*-factors(gt) *etc*. and is not relevant to the choice of reflections for refinement. *R*-factors based on *F*^2^ are statistically about twice as large as those based on *F*, and *R*- factors based on ALL data will be even larger.

### Fractional atomic coordinates and isotropic or equivalent isotropic displacement parameters (Å^2^)

**Table:**

	*x*	*y*	*z*	*U*_iso_*/*U*_eq_	
C1	0.25132 (14)	0.6495 (2)	0.51893 (9)	0.0385 (3)	
N1	0.34516 (13)	0.7171 (2)	0.59164 (8)	0.0450 (3)	
C2	0.12494 (14)	0.6840 (2)	0.47750 (9)	0.0370 (3)	
N2	0.09944 (13)	0.7886 (2)	0.51157 (8)	0.0442 (3)	
C3	0.19586 (15)	0.8590 (2)	0.58719 (9)	0.0408 (3)	
C4	0.17061 (19)	0.9715 (3)	0.62465 (12)	0.0566 (5)	
H4	0.0893	0.9985	0.5973	0.068*	
C5	0.2658 (2)	1.0397 (3)	0.70076 (12)	0.0597 (5)	
H5	0.2490	1.1132	0.7253	0.072*	
C6	0.3897 (2)	1.0000 (3)	0.74291 (12)	0.0583 (5)	
H6	0.4537	1.0465	0.7952	0.070*	
C7	0.41593 (18)	0.8936 (3)	0.70727 (11)	0.0537 (5)	
H7	0.4978	0.8684	0.7352	0.064*	
C8	0.31893 (15)	0.8217 (2)	0.62808 (9)	0.0410 (3)	
C9	0.28488 (14)	0.5376 (2)	0.48137 (9)	0.0413 (4)	
C10	0.27772 (17)	0.3643 (3)	0.48187 (11)	0.0501 (4)	
C11	0.30563 (18)	0.2628 (3)	0.44390 (13)	0.0590 (5)	
H11	0.3005	0.1466	0.4446	0.071*	
C12	0.34054 (19)	0.3366 (3)	0.40569 (13)	0.0647 (6)	
H12	0.3583	0.2700	0.3798	0.078*	
C13	0.3498 (2)	0.5088 (3)	0.40510 (14)	0.0652 (6)	
H13	0.3744	0.5565	0.3792	0.078*	
C14	0.32275 (16)	0.6126 (3)	0.44284 (12)	0.0524 (4)	
H14	0.3295	0.7285	0.4426	0.063*	
Cl1	0.23549 (8)	0.27027 (8)	0.53138 (5)	0.0854 (2)	
C15	0.01547 (14)	0.6069 (2)	0.39677 (9)	0.0379 (3)	
C16	0.00943 (14)	0.5764 (2)	0.33031 (10)	0.0396 (3)	
H16	0.0777	0.6000	0.3370	0.047*	
C17	−0.09655 (14)	0.5118 (2)	0.25514 (10)	0.0397 (3)	
C18	−0.20106 (15)	0.4789 (2)	0.24403 (10)	0.0422 (4)	
C19	−0.19425 (16)	0.5068 (2)	0.30999 (11)	0.0474 (4)	
H19	−0.2622	0.4826	0.3036	0.057*	
C20	−0.08736 (16)	0.5706 (2)	0.38564 (11)	0.0457 (4)	
H20	−0.0847	0.5891	0.4291	0.055*	
O1	−0.10954 (11)	0.47659 (19)	0.18770 (8)	0.0534 (3)	
C21	−0.0123 (2)	0.5263 (3)	0.19222 (14)	0.0655 (6)	
H21A	0.0016	0.6447	0.2027	0.098*	
H21B	−0.0346	0.5012	0.1402	0.098*	
H21C	0.0618	0.4667	0.2368	0.098*	
O2	−0.30319 (12)	0.42185 (19)	0.16651 (8)	0.0567 (4)	
C22	−0.4155 (2)	0.4121 (4)	0.14824 (15)	0.0743 (7)	
H22A	−0.4075	0.3313	0.1845	0.111*	
H22B	−0.4808	0.3785	0.0910	0.111*	
H22C	−0.4345	0.5199	0.1568	0.111*	

### Atomic displacement parameters (Å^2^)

**Table:**

	*U*^11^	*U*^22^	*U*^33^	*U*^12^	*U*^13^	*U*^23^
C1	0.0357 (7)	0.0417 (8)	0.0320 (7)	0.0051 (6)	0.0204 (6)	0.0026 (6)
N1	0.0359 (6)	0.0552 (9)	0.0349 (6)	0.0019 (6)	0.0203 (6)	−0.0007 (6)
C2	0.0338 (7)	0.0411 (8)	0.0293 (6)	0.0050 (6)	0.0186 (6)	0.0025 (6)
N2	0.0368 (6)	0.0536 (8)	0.0321 (6)	0.0080 (6)	0.0193 (5)	−0.0007 (6)
C3	0.0417 (8)	0.0429 (8)	0.0317 (7)	0.0051 (6)	0.0223 (6)	0.0019 (6)
C4	0.0561 (11)	0.0628 (12)	0.0428 (9)	0.0140 (9)	0.0302 (9)	−0.0030 (8)
C5	0.0749 (13)	0.0549 (11)	0.0437 (9)	0.0048 (10)	0.0378 (10)	−0.0061 (8)
C6	0.0662 (12)	0.0590 (12)	0.0391 (8)	−0.0155 (10)	0.0315 (9)	−0.0108 (8)
C7	0.0441 (9)	0.0661 (12)	0.0392 (8)	−0.0103 (8)	0.0235 (7)	−0.0077 (8)
C8	0.0394 (8)	0.0446 (8)	0.0327 (7)	−0.0015 (6)	0.0218 (6)	0.0007 (6)
C9	0.0316 (7)	0.0486 (9)	0.0331 (7)	0.0069 (6)	0.0176 (6)	0.0021 (6)
C10	0.0454 (9)	0.0512 (10)	0.0430 (8)	0.0113 (8)	0.0256 (7)	0.0072 (7)
C11	0.0497 (10)	0.0554 (11)	0.0498 (10)	0.0138 (8)	0.0248 (9)	−0.0027 (8)
C12	0.0530 (11)	0.0824 (16)	0.0535 (11)	0.0104 (10)	0.0339 (10)	−0.0119 (10)
C13	0.0634 (12)	0.0839 (16)	0.0655 (13)	−0.0011 (11)	0.0504 (11)	−0.0087 (11)
C14	0.0441 (9)	0.0675 (12)	0.0512 (10)	0.0010 (8)	0.0345 (8)	−0.0038 (9)
Cl1	0.1261 (6)	0.0590 (4)	0.1054 (5)	0.0107 (3)	0.0921 (5)	0.0207 (3)
C15	0.0331 (7)	0.0407 (8)	0.0318 (7)	0.0055 (6)	0.0187 (6)	0.0020 (6)
C16	0.0323 (7)	0.0445 (8)	0.0358 (7)	0.0019 (6)	0.0206 (6)	−0.0008 (6)
C17	0.0379 (7)	0.0402 (8)	0.0350 (7)	0.0024 (6)	0.0222 (6)	−0.0015 (6)
C18	0.0361 (7)	0.0378 (8)	0.0403 (8)	−0.0029 (6)	0.0208 (6)	−0.0038 (6)
C19	0.0407 (8)	0.0528 (10)	0.0490 (9)	−0.0066 (7)	0.0305 (8)	−0.0023 (8)
C20	0.0433 (8)	0.0528 (10)	0.0413 (8)	0.0004 (7)	0.0287 (7)	0.0002 (7)
O1	0.0455 (7)	0.0715 (9)	0.0404 (6)	−0.0064 (6)	0.0279 (6)	−0.0139 (6)
C21	0.0643 (12)	0.0865 (16)	0.0571 (11)	−0.0110 (11)	0.0456 (11)	−0.0154 (11)
O2	0.0420 (6)	0.0675 (9)	0.0488 (7)	−0.0163 (6)	0.0259 (6)	−0.0196 (6)
C22	0.0475 (11)	0.0965 (19)	0.0659 (13)	−0.0286 (11)	0.0332 (10)	−0.0219 (13)

### Geometric parameters (Å, °)

**Table:**

C1—N1	1.318 (2)	C12—H12	0.9300
C1—C2	1.438 (2)	C13—C14	1.397 (3)
C1—C9	1.499 (2)	C13—H13	0.9300
N1—C8	1.371 (2)	C14—H14	0.9300
C2—N2	1.325 (2)	C15—C20	1.388 (2)
C2—C15	1.490 (2)	C15—C16	1.405 (2)
N2—C3	1.367 (2)	C16—C17	1.383 (2)
C3—C8	1.403 (2)	C16—H16	0.9300
C3—C4	1.418 (2)	C17—O1	1.371 (2)
C4—C5	1.362 (3)	C17—C18	1.407 (2)
C4—H4	0.9300	C18—O2	1.368 (2)
C5—C6	1.411 (3)	C18—C19	1.384 (3)
C5—H5	0.9300	C19—C20	1.389 (2)
C6—C7	1.367 (3)	C19—H19	0.9300
C6—H6	0.9300	C20—H20	0.9300
C7—C8	1.414 (2)	O1—C21	1.419 (3)
C7—H7	0.9300	C21—H21A	0.9600
C9—C10	1.386 (3)	C21—H21B	0.9600
C9—C14	1.412 (3)	C21—H21C	0.9600
C10—C11	1.400 (3)	O2—C22	1.412 (3)
C10—Cl1	1.732 (2)	C22—H22A	0.9600
C11—C12	1.368 (3)	C22—H22B	0.9600
C11—H11	0.9300	C22—H22C	0.9600
C12—C13	1.381 (4)		
			
N1—C1—C2	122.11 (15)	C12—C13—C14	121.0 (2)
N1—C1—C9	115.67 (14)	C12—C13—H13	119.5
C2—C1—C9	122.21 (13)	C14—C13—H13	119.5
C1—N1—C8	117.75 (14)	C13—C14—C9	118.5 (2)
N2—C2—C1	120.19 (14)	C13—C14—H14	120.7
N2—C2—C15	115.39 (13)	C9—C14—H14	120.7
C1—C2—C15	124.41 (14)	C20—C15—C16	118.62 (14)
C2—N2—C3	118.32 (14)	C20—C15—C2	118.11 (14)
N2—C3—C8	121.09 (15)	C16—C15—C2	123.21 (14)
N2—C3—C4	119.23 (16)	C17—C16—C15	121.01 (15)
C8—C3—C4	119.69 (16)	C17—C16—H16	119.5
C5—C4—C3	119.79 (19)	C15—C16—H16	119.5
C5—C4—H4	120.1	O1—C17—C16	124.67 (15)
C3—C4—H4	120.1	O1—C17—C18	115.51 (14)
C4—C5—C6	120.75 (19)	C16—C17—C18	119.82 (15)
C4—C5—H5	119.6	O2—C18—C19	125.31 (16)
C6—C5—H5	119.6	O2—C18—C17	115.67 (16)
C7—C6—C5	120.32 (17)	C19—C18—C17	119.02 (15)
C7—C6—H6	119.8	C18—C19—C20	121.00 (16)
C5—C6—H6	119.8	C18—C19—H19	119.5
C6—C7—C8	120.12 (18)	C20—C19—H19	119.5
C6—C7—H7	119.9	C15—C20—C19	120.50 (16)
C8—C7—H7	119.9	C15—C20—H20	119.7
N1—C8—C3	120.49 (14)	C19—C20—H20	119.7
N1—C8—C7	120.19 (16)	C17—O1—C21	117.50 (14)
C3—C8—C7	119.31 (17)	O1—C21—H21A	109.5
C10—C9—C14	119.28 (17)	O1—C21—H21B	109.5
C10—C9—C1	122.33 (16)	H21A—C21—H21B	109.5
C14—C9—C1	118.37 (16)	O1—C21—H21C	109.5
C9—C10—C11	121.20 (19)	H21A—C21—H21C	109.5
C9—C10—Cl1	119.82 (15)	H21B—C21—H21C	109.5
C11—C10—Cl1	118.97 (17)	C18—O2—C22	117.59 (16)
C12—C11—C10	119.1 (2)	O2—C22—H22A	109.5
C12—C11—H11	120.4	O2—C22—H22B	109.5
C10—C11—H11	120.4	H22A—C22—H22B	109.5
C11—C12—C13	120.9 (2)	O2—C22—H22C	109.5
C11—C12—H12	119.6	H22A—C22—H22C	109.5
C13—C12—H12	119.6	H22B—C22—H22C	109.5

## References

[bb1] Bellizzi, M., Crundwell, G., Zeller, M., Hunter, A. D. & McBurney, B. (2006). *Acta Cryst.* E**62**, o5249–o5251.

[bb2] Cantalupo, S., Salvati, H., McBurney, B., Raju, R., Glagovich, N. & Crundwell, G. (2006). *J. Chem. Crystallogr.* **36**, 17–24.

[bb3] Crundwell, G. & Stacy, V. (2005). *Acta Cryst.* E**61**, o3159–o3160.

[bb4] Farrugia, L. J. (1997). *J. Appl. Cryst.* **30**, 565.

[bb5] Fitchett, C. M. & Steel, P. J. (2006). *Dalton Trans.* pp. 4886–4888. 10.1039/b611622c 17047736

[bb6] Oxford Diffraction (2009). *CrysAlis CCD*, *CrysAlis PRO* and *CrysAlis RED* Oxford Diffraction Ltd, Yarnton, England.

[bb7] Patra, G. K., Goldberg, I., De, S. & Datta, D. (2007). *CrystEngComm*, **9**, 828–832.

[bb8] Sheldrick, G. M. (2008). *Acta Cryst.* A**64**, 112–122. 10.1107/S0108767307043930 18156677

